# Characterization of air flow and lung function in the pulmonary acinus by fluid-structure interaction in idiopathic interstitial pneumonias

**DOI:** 10.1371/journal.pone.0214441

**Published:** 2019-03-28

**Authors:** Long Chen, Xia Zhao

**Affiliations:** 1 College of Aerospace Engineering, Nanjing University of Aeronautics and Astronautics, Nanjing, Jiangsu, China; 2 Department of Rheumatology, Affiliated Hospital of Nanjing University of Chinese Medicine, Nanjing, Jiangsu, China; Technion Israel Institute of Technology, ISRAEL

## Abstract

**Background and objective:**

The idiopathic interstitial pneumonias (IIPs) are diffuse parenchymal lung disorders that are associated with substantial morbidity and mortality. Early diagnosis and disease stratification of IIP patients are important because these are related with the treatment and prognosis. Idiopathic pulmonary fibrosis (IPF) and nonspecific interstitial pneumonia (NSIP) are two major distinctive pathologic patterns of pulmonary fibrosis. We researched the application of the fluid-structure interaction (FSI) to the respiratory system and compared the pulmonary acinus mechanics and functions in healthy and IIP models.

**Methods:**

The human pulmonary alveolus is idealized by a three-dimensional honeycomb-like geometry, and a fluid-structure interaction analysis is performed to study the normal and diseased breathing mechanics. The computational domain consists of two generations of alveolar ducts within the pulmonary acinus, with alveolar geometries approximated as closely packed 14-sided polygons.

**Findings:**

In a normal breathing cycle, the flow rate of the healthy model is significantly larger than that of the NSIP and IPF models. Similar trends are observed for the volume change and the maximum pressure drop. The flow rate and the volume change of the NSIP are almost the same as those of IPF. The maximum pressure drop of NSIP is 5.5% larger than that of IPF. There is a 47% decrease in the pulmonary acinus compliance for the NSIP and IPF compared with that of the healthy model. The acinus resistances of NSIP and IPF are higher than those of the healthy lung by 6.4~11.2%. In particular, the pulmonary acinus resistance of the NSIP lung is higher than that of the IPF lung by 4.5%.

**Conclusions:**

Our study demonstrates the differences of air flow and lung function in the pulmonary acinus between the healthy and the IIP models. These changes in the lung are important considerations for early diagnosis and disease stratification in patients. Patient-based geometry can to be included in the computational models in future studies.

## Introduction

Idiopathic interstitial pneumonias (IIPs) are a group of diffuse lung diseases that share many similar radiologic and pathologic features. IIPs are associated with significant morbidity and mortality. The classification of IIPs was revised by the American Thoracic Society and the European Respiratory Society (ATS/ERS) in 2013, and three categories, which include major IIPs, rare IIPs and unclassifiable models, were created [1]. Among these, chronic fibrosing in major IIPs is mainly divided into two groups in pathology: idiopathic pulmonary fibrosis (IPF) and nonspecific interstitial pneumonia (NSIP). IPF is a chronic lung disease with a severe prognosis and unknown pathogenesis that is associated with the histopathologic and/or radiologic pattern of the usual interstitial pneumonia (UIP) pattern [2]. Patients with NSIP often have a good response to corticosteroids, while patients with IPF can worsen on corticosteroids [3] and are currently treated with anti-fibrotic agents [4, 5]. How to separate and classify differential pathologic patterns of IIPs in a clinical study becomes a perplexing problem, because in clinical practice, surgical biopsy rates are low due to concerns regarding associated morbidity and mortality [6]. Pathologic patterns previously formed the basis for the classification of IIP subtypes, whereas in the updated classification, greater emphasis is given for multidisciplinary diagnoses to improve precision medicine [7].

Fluid-structure interaction (FSI) is the interaction of some movable or deformable structure with an internal or surrounding fluid flow [8]. FSI occurs in many innate biomechanical functions in the human body, such as blood flow and air flow through lungs and the pulmonary alveolus. Many studies have demonstrated the applicability of FSI analysis in these areas. Bavo et al. used immersed-boundary-based FSI (IB-FSI) [9] and arbitrary Lagrangian-Eulerian-based FSI (ALE-FSI) methods to simulate prosthetic aortic valves. Kim et al. used a FSI method to study aging effects on airflow dynamics [10]. Xia et al. used a 3D FSI method to simulate the flow at different Reynolds numbers and airway wall stiffness [11]. A FSI analysis in a 2D model of alveolar sacs was conducted by Dailey and Ghadiali [12]. However, so far there is no FSI simulation in pulmonary alveolus during normal breathing in IIPs.

The overall performance of the lung was controlled by the mechanics of its microstructure. However, the alveolar structure was too small to allow direct mechanical measurements. Fung formed an alveolar ductal tree by 14-hedrons (truncated octahedral) [13]. Denny and Schroter used an assemblage of 14-hedrons to represent an alveolar duct unit for finite element analysis [14]. Yan et al. connected 14-hedrons to form a space-filling air space by removing some of the interconnecting faces and, thus, formed alveolar openings or ducts [15]. Following in these footsteps, the present alveolus geometry composed of 14-hedrons to a 3D honeycomb-like geometry obtained from a scanning electron micrograph (SEM), and more detailed descriptions can be found in study by Dutta et al. and Haefeli-Bleuer et al. [16, 17].

Healthy lung is characterized by thin septal and alveolar walls with a thin pleural space. The pathologic pattern of NSIP shows diffuse involvement of the alveolar walls with thickening, fusion and simplification [18]. In some areas, there is more significant fusion and thickening, which lead to a suggestion of heterogeneity, despite its being a diffuse process [19]. The pathologic pattern of IPF is marked with irregular fibrosis in a peripheral and subpleural distribution. The fibrosis transitions to completely normal alveolar walls abruptly, with numerous fibroblast foci at the interface zone. In some lobules, microscopic honeycombing change occurred. The illustrations of a healthy lung, NSIP and IPF highlight the key schematic representation and pathology of the patterns of pulmonary fibrosis [20]. In addition, the illustrations also allow comparisons between the pathologic and radiologic patterns [21].

In this study, the human pulmonary alveolus is idealized by a three-dimensional honeycomb-like geometry, and a fluid-structure analysis is performed to study the normal breathing mechanics in healthy and pulmonary fibrosis models. In this model, we apply a negative pressure on the outside surface of the pulmonary acinus, which causes air to flow in and out of the pulmonary acinus, which simulates normal breathing. This model provides a useful tool for predicting breathing mechanics and for comparing the differences between healthy and pulmonary fibrosis (NSIP and IPF) models.

## Materials and methods

### Geometric models

Our simulation starts from the geometric model at the resting state of the breathing cycle. Each alveolar sac contains 12 alveolar spaces shaped as 14-hedrons. The alveolar duct is developed based on anatomical model dimensions [22, 23]. The dimension of the healthy model is scaled to the functional residual capacity (FRC) of 3.45 L [24]. FRC is the resting volume of the air present in the lungs at the end of normal expiration. The total length for the alveolar sacs is 1 mm. The characteristic diameter for the alveolar duct is 0.274 mm [22, 23]. The mechanical properties of tissue and the thickness of tissue layers for the healthy model are determined based on the study by Kim [25]. The tissue thickness of the healthy model is 0.025 mm which was employed from the references [26–28]. The equivalent small strain modulus of the tissue is 35,714 Pa [25]. The Poisson’s ratio of the tissue is 0.42 [11]. The 3D geometry models are shown in the S1 File.

**Fig 1:**
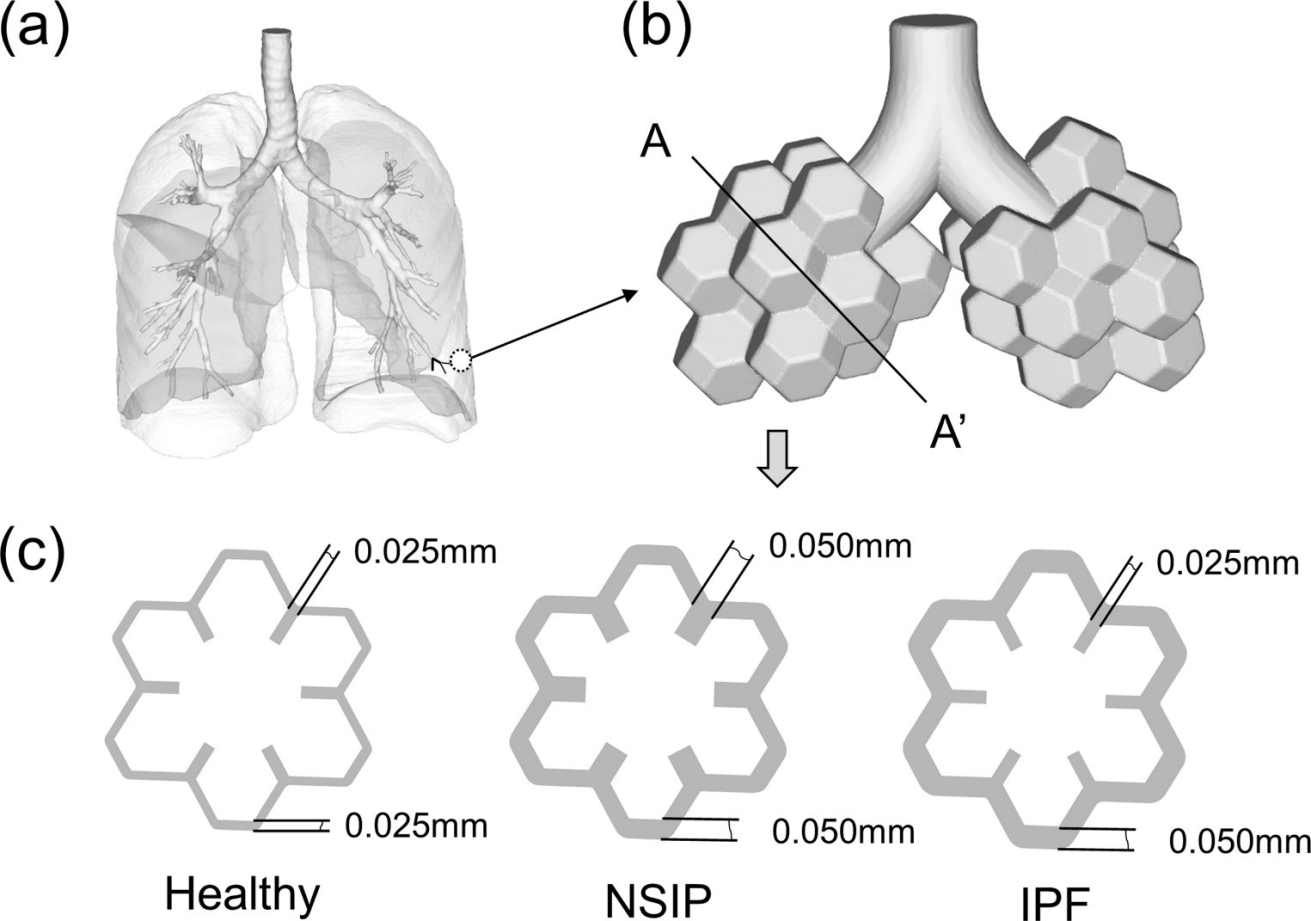
Geometric models of pulmonary acinus. (a) Human airway model. (b) Two generations of alveolar ducts within the pulmonary acinus and alveolar sac composed of 14-hedrons. (c) Cross-section of alveolar sac for healthy, NSIP, and IPF models.

According to the pathological and radiological features of healthy, NISP and IPF lungs [5, 29], geometric models of the pulmonary acinus were built for FSI simulations, as shown in Fig 1. The computed tomography (CT)-based respiratory tree for a whole lung is shown in Fig 1A.

A pulmonary alveolus is a hollow cavity that is found in the lung parenchyma, and it is the end of the respiratory tree. Pulmonary alveoli connect with the alveolar sac. Pulmonary acinus includes the alveolar sac, alveolar and other distal lung tissue, as shown in Fig 1B. It is the basic functional unit of the lung. A cross-section of the alveolar sac for healthy, NSIP, and IPF models shown in Fig 1C accords with the schematic representation of the patterns of pulmonary fibrosis and a healthy lung. Parameters of the primary septa thickness, secondary septa thickness, equivalent small strain modulus, resting volume, and tidal volume (TV) of the three models are listed as Table 1. The parameters of primary and secondary septa thickness of healthy model were employed from the references [26–28]. We set the TVs and the degree of thickening of the NSIP and IPF models to be equal because lung function tests do not easily distinguish between NSIP and IPF. Based on this assumption, the volume of each model is shown in Table 1.

**Table 1:** Parameters for healthy, NSIP, and IPF models.

	Primary septa thickness (mm)	Secondary septa thickness (mm)	Equivalent small strain modulus (Pa)	Resting Volume (mm^3^)	Tidal volume (mm^3^)*
**Healthy**	0.025	0.025	35,714	0.512	0.0766
**NSIP**	0.050	0.050	35,714	0.493	0.0374
**IPF**	0.050	0.025	35,714	0.465	0.0371

*Tidal volumes are calculated by fluid-structure interaction simulation, and more detailed descriptions can be found in the results section.

### Computational models

#### Governing equations

The governing equations for airflow in the fluid domain are the time-dependent incompressible Navier-Stokes equations.

$$\frac{\partial u_{j}}{\partial x_{j}}=0$$ (1)

ρ∂ui∂t+ρ∂ujui∂xj+∂p∂xi=μ∂2ui∂xj∂xi (2)

where *u*_*j*_ is the air velocity, *ρ* is the density of fluid, *p* is the pressure, and *μ* is the dynamic viscosity. The Navier-Stokes equations were solved numerically on a moving grid using an open-source computational fluid dynamics (CFD) solver, Nalu [30] (https://github.com/NaluCFD). Nalu is a generalized unstructured massively parallel low Mach flow code that was designed to support a variety of open applications. This code base began as an effort to prototype the Sierra Toolkit [31] usage along with direct parallel matrix assembly to the Trilinos [32] data structure. Nalu has evolved as a tool to support a variety of low speed research projects.

For all simulations, a time step size of 0.01 s and second-order accuracy in time discretization was used (Comparison of relative volume change at different time steps is shown in the S1 Fig). This time step agrees with the value chosen by Kim et al. [10]. Muelu [32] was used as the preconditioner of linear solvers. The relative tolerance used to determine the convergence of the linear system was 10^−5^. (Example of Nalu input file is shown in the S2 File.) According to the results section, the maximum Reynolds number based on the alveolar sac lengths is 0.08. Hence, the assumption of laminar flow is assumed in the simulation.

Tissue deformation in the solid domain is governed by time-dependent structural equations.

$$\rho \frac{\partial^{2}d_{i}}{\partial t^{2}}=\frac{\partial \sigma_{ij}}{\partial x_{j}}+f_{i}$$ (3)

$$\sigma_{ij}=C_{ijkl}\varepsilon_{kl}$$ (4)

where *d*_*i*_ is the displacement, *σ*_*ij*_ is the Cauchy stress tensor, *ρ* is the density of tissue, *f*_*i*_ is the external force, *C*_*ijkl*_ is the elasticity tensor, and *ε*_*kl*_ is the infinitesimal strain tensor. An in-house computational structural dynamics (CSD) solver FEAP [33] (http://projects.ce.berkeley.edu/feap) was used to solve the structural equations. FEAP is a general purpose finite element analysis program that was designed for research and educational use. For parallel simulation, the Super_LU package [34] was used to solve the liner equations.

The pulmonary acinus tissue is assumed to be a hyper-elastic material in the non-linear material models. Among these models, the Neo-Hookean model is used for the tissue [35, 36]. In the FEAP code, the modified Neo-Hookean model is used, with the stored energy function is given as

$$W=KU\left(J\right)+\frac{1}{2}G\left(J^{-2/3}I_{C}-3\right)$$ (5)

where the parameters *K* and *G* are equivalent to the small strain bulk and shear moduli, respectively. $U(J)=1/4\left(J^{2}-1-2\text{ln}J\right)$ is the volumetric deformation function, J is the determinant of the deformation gradient, and *I*_*C*_ is the first principal invariant of the deformation tensor. The FEAP input data for the model is specified in terms of the equivalent small strain modulus (E) and Poisson’s ratio (ν) such that the K and G are given by

$$K=\frac{E}{3(1-2\nu )}G=\frac{E}{2(1+\nu )}$$ (6)

#### Fluid-structure interaction

**Fig 2:**
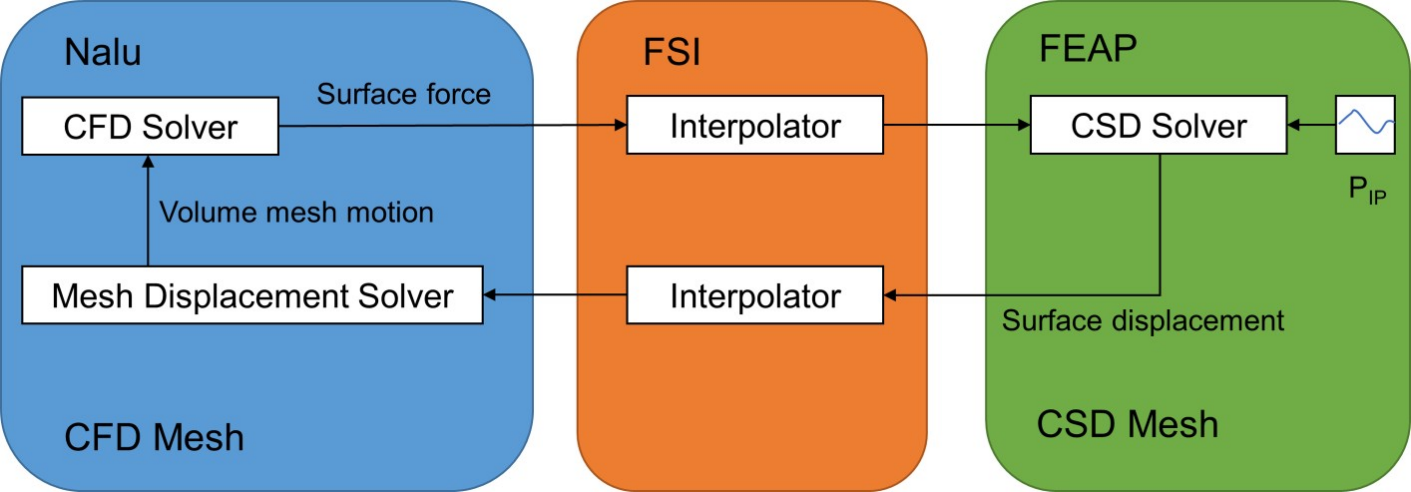
A schematic diagram of FSI iteration loop. FSI coupling package is developed in the Python language (the top level FSI iteration script is shown in the S3 File). FSI coupling package and solvers exchange data of displacement and force using MPI.

A two-way coupling between the fluid and structure domain is used to solve the governing equations in an iterative manner, as shown in Fig 2. In the normal breathing cycle, the simulation starts from the resting volume. The intrapleural pressure load is applied on the outer surface of the tissue. The structural equations are solved, which provide deformation at the fluid-structure interface. The volume mesh of fluid is moved by the solver of the mesh displacement equations in accordance with the deformation of the fluid-structure interface. The Navier-Stokes equations are solved for the fluid domain based on the moved mesh, and then the fluid forces are computed on the structure surface. The fluid forces are applied on the inner surface of the tissue for the next time step. At the fluid structure interface, the two meshes are conformed to each other. Usually, different surface meshes are applied on the two domains of the interface. A radial basis functions (RBF) [37] based interpolation method is used to interpolate the displacements from the structure mesh to the fluid mesh and interpolate forces from the fluid mesh to the structure mesh. The construction of the FSI method is implemented by coupling Nalu with FEAP. Nalu was written in the C++ language, and FEAP was written in the Fortran language. For this reason, a FSI coupling package was developed in the Python language for the FSI iteration control (the top level FSI iteration script is shown in the S3 File). The FSI coupling package and solvers exchanged the data on displacement and force using the message passing interface (MPI).

**Fig 3:**
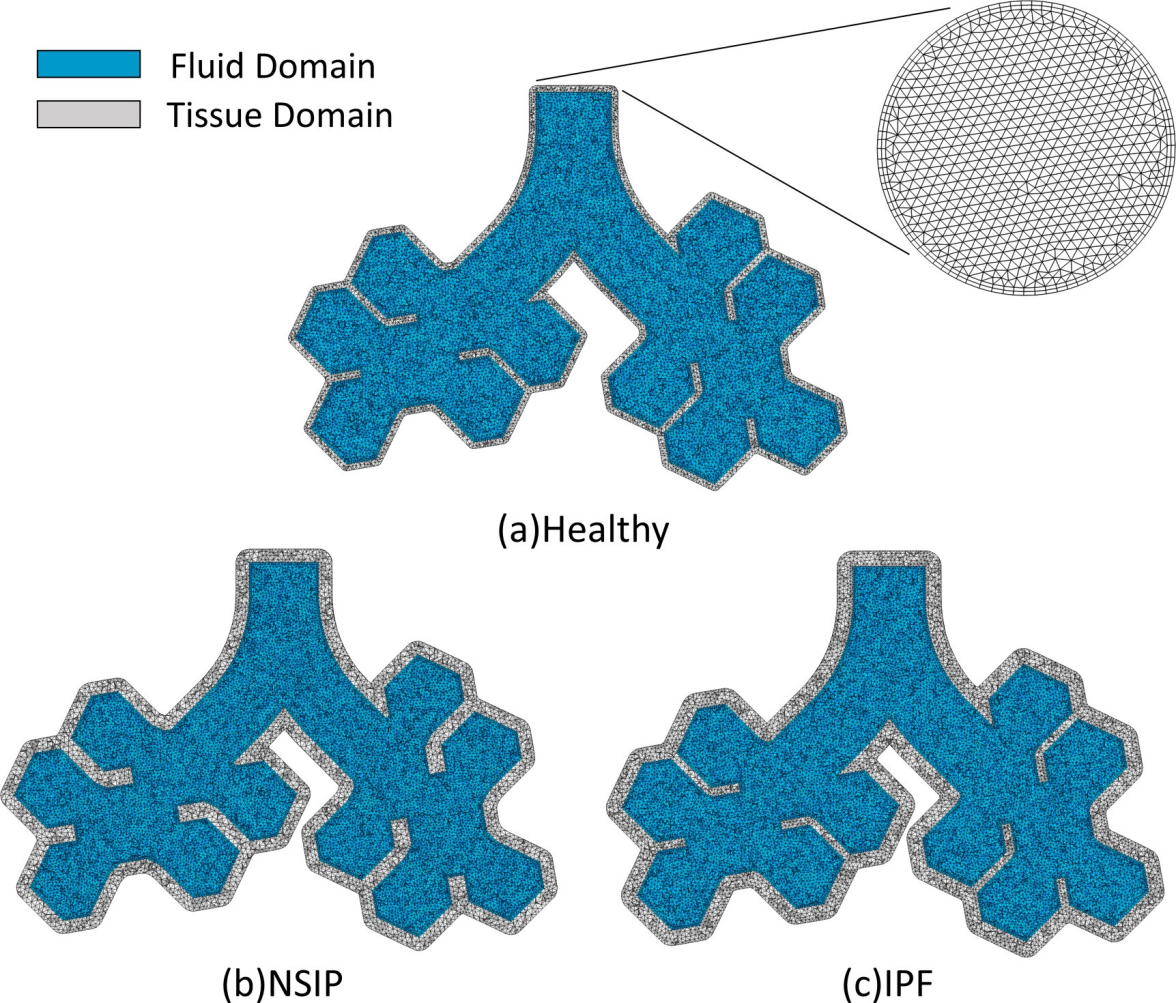
Cross-section and open boundary surface of computational meshes for fluid domain and tissue domain. (a) Healthy, (b) NSIP, and (c) IPF.

The CFD and CSD meshes used are shown in Fig 3. For each fluid domain, hybrid unstructured meshes composed of tetrahedrons and prims are generated as CFD meshes. Two layers of prism elements are adopted near the wall to ensure accurate resolution of the boundary layer[38]. For each tissue domain, unstructured meshes composed of tetrahedrons are generated. To ensure independence of the computational grid used, a mesh convergence study is performed. (Mesh convergence study parameters are shown in the S1 Table.) The CFD and CSD meshes sized for the present simulation are shown in Table 2. All computations are performed on a workstation with Intel Xeon processors. The central processing unit (CPU) time is approximately 12 hours on 28 processors for one cycle of unsteady simulation.

**Table 2:** CFD and CSD mesh sizes.

	Healthy	NSIP	IPF
**CFD mesh elements**	2.31M	2.46M	2.54M
**CSD mesh elements**	0.99M	1.18M	1.22M

#### Boundary conditions

**Fig 4:**
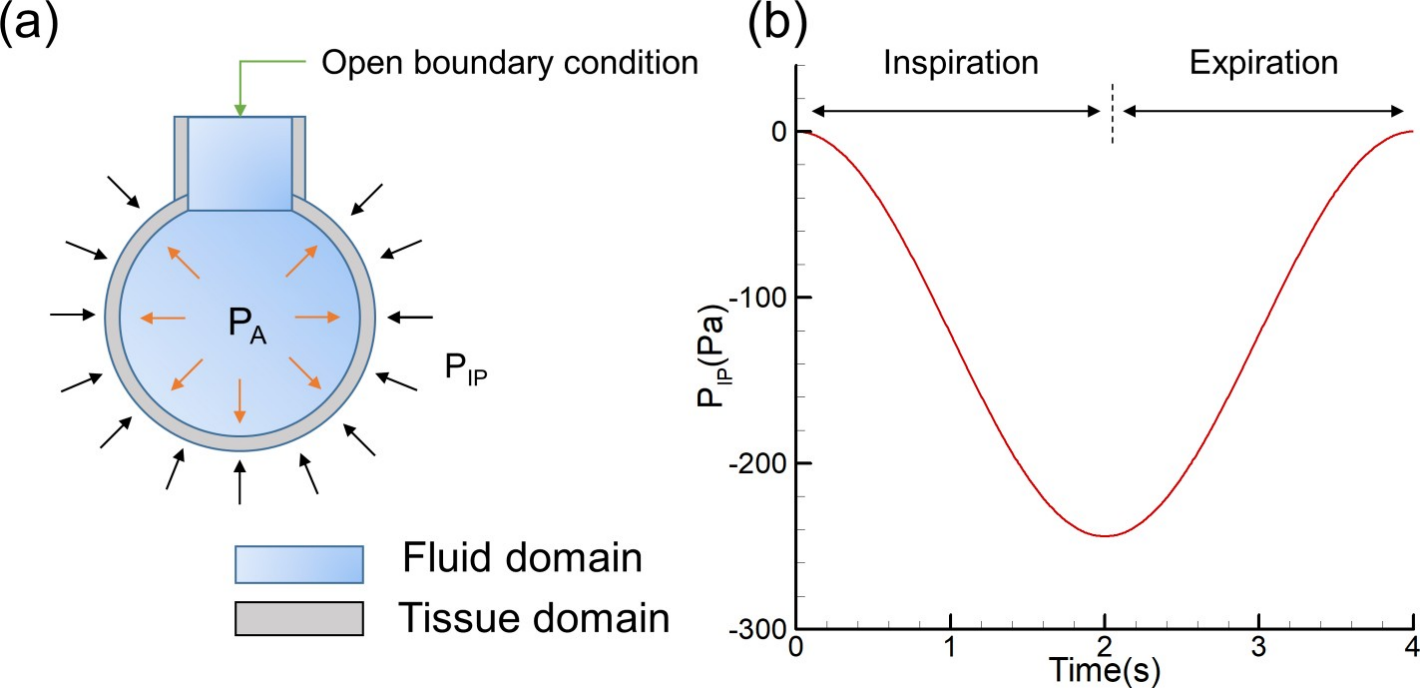
Model schematic showing boundary conditions and applied loads. (a) Pulmonary acinus model boundary conditions. P_A_ is pressure in the alveolar and is applied to the inner surface of the tissue. P_IP_ is intrapleural pressure and is applied to the outer surface of the tissue. (b) Intrapleural pressure ranging from 0 (FRC) to -244 Pa (FRC+TV).

Negative pressure in the pleural cavity plays an important role in normal respiration. During inhalation, the inspiratory muscles contract and the ribcage moves up and out while the diaphragm moves down. Tissue stresses are transferred through the lung parenchyma and increase the transmural pressure gradient. Air flows into the lung. During exhalation, inspiratory muscles relax, and the elastic recoil decreases the alveolar volume and increases the alveolar air pressure. Air flows out of the lung. Therefore, at the alveolar level, tissue deformation drives the flow field. A sinusoidally pressure ranging from 0 (FRC) to -2.5 cmH_2_O (FRC+TV) is adopted to represent the parenchymal tethering for normal breathing within a 4 s breathing period [39], as shown in Fig 4. According to this pressure profile, the intrapleural pressure can be approximately expressed as:

$$p_{IP}=-p_{0}\left[\frac{1}{2}-\frac{1}{2}\cos\left(\omega t\right)\right]$$ (7)

$$p_{0}=244Pa\approx 2.5cmH_{2}O$$ (8)

$$\omega =\frac{\pi }{2}1/s$$ (9)

where *p* represents the pressure amplitude and *ω* represents the circular frequency. No-slip boundary consideration was assumed at the fluid-solid interface, implying that there is no relative velocity between the fluid and the solid mesh boundaries. The open boundary condition is defined by a zero static pressure. In contrast to the study using an inlet or an outlet boundary condition [16] in which the flow rate is predefined, the air can flow in and out of the pulmonary acinus from the open boundary condition based on the pressure difference.

## Results

Results of pulmonary acinus mechanics (pressure drop, displacement, velocity, and maximum principal stress) and pulmonary acinus function (flow rate, volume, pressure-volume loop, flow-volume loop, and pulmonary acinus resistance) for healthy, NSIP and IPF models are obtained from the computational simulation and are discussed below.

### Pulmonary acinus mechanics

**Fig 5:**
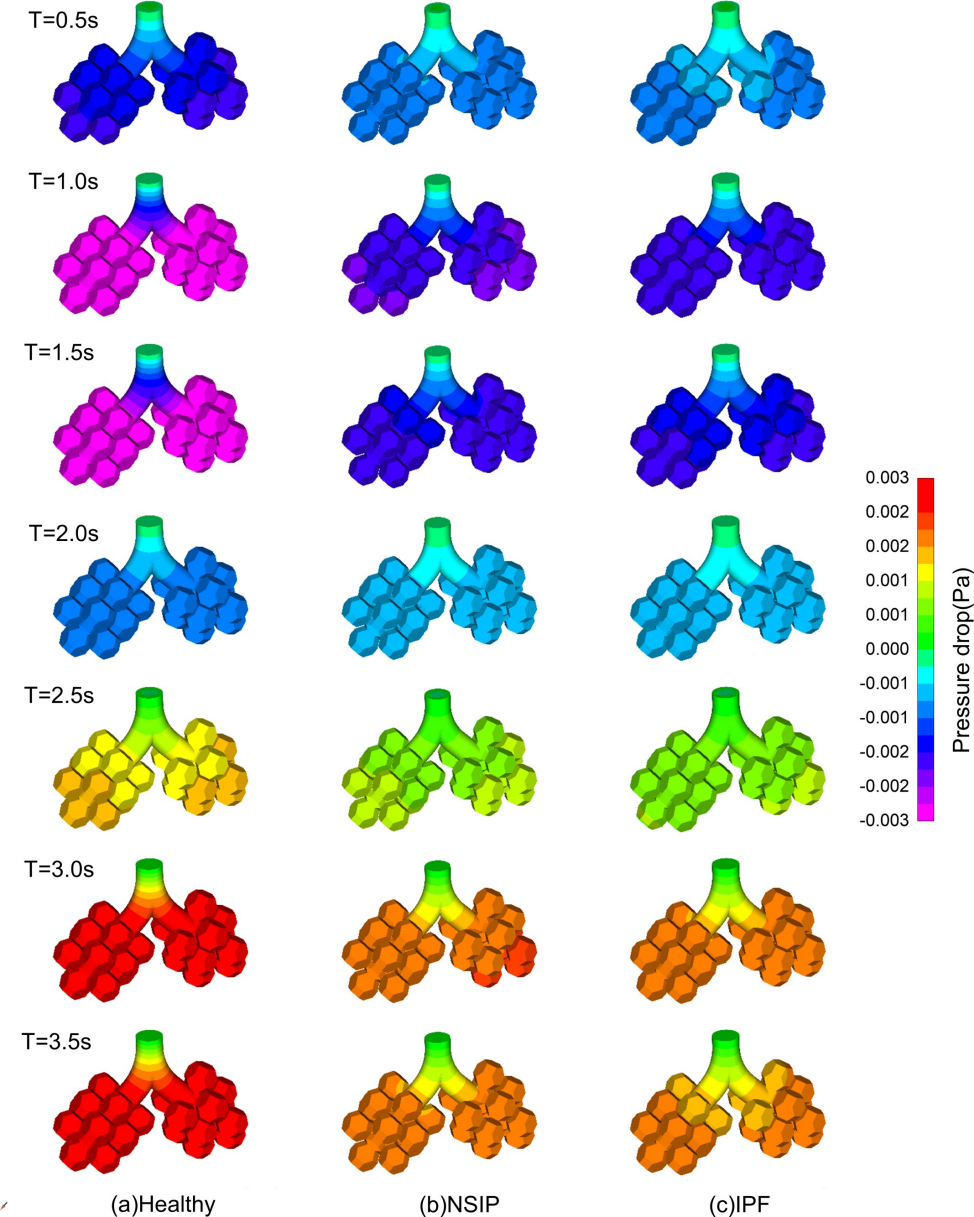
Temporal variation in contour of pressure drop at T = 0.5 s, T = 1.0 s, T = 1.5 s, T = 2.0 s, T = 2.5 s, T = 3.0 s, T = 3.5 s. (a) Healthy, (b) NSIP, and (c)IPF.

**Fig 6:**
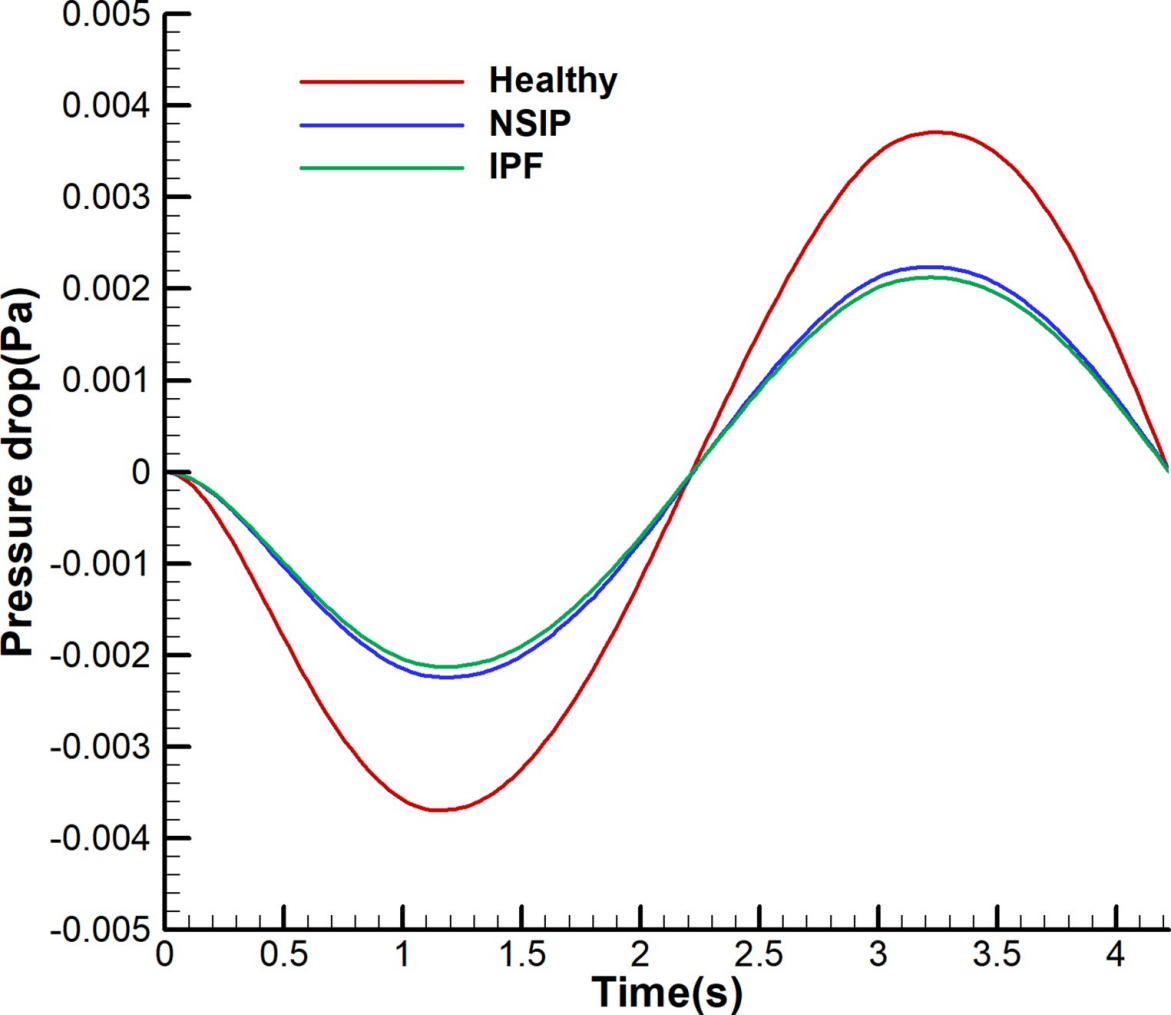
Maximum pressure drop history for healthy, NSIP, and IPF models.

Fig 5 shows that the pressure drop varied during inspiration and expiration, and the magnitude of the pressure drop of NSIP is larger than that of IPF. During inspiration, tissue deformation drives air flow into the pulmonary acinus, and the pressure drop is negative. During expiration, tissue deformation drives air flow out of the pulmonary acinus, and the pressure drop is positive. For specific data, as shown in Fig 6, the maximum pressure drop decreases by 43% for the NSIP and IPF models compared with that of the healthy model.

**Fig 7:**
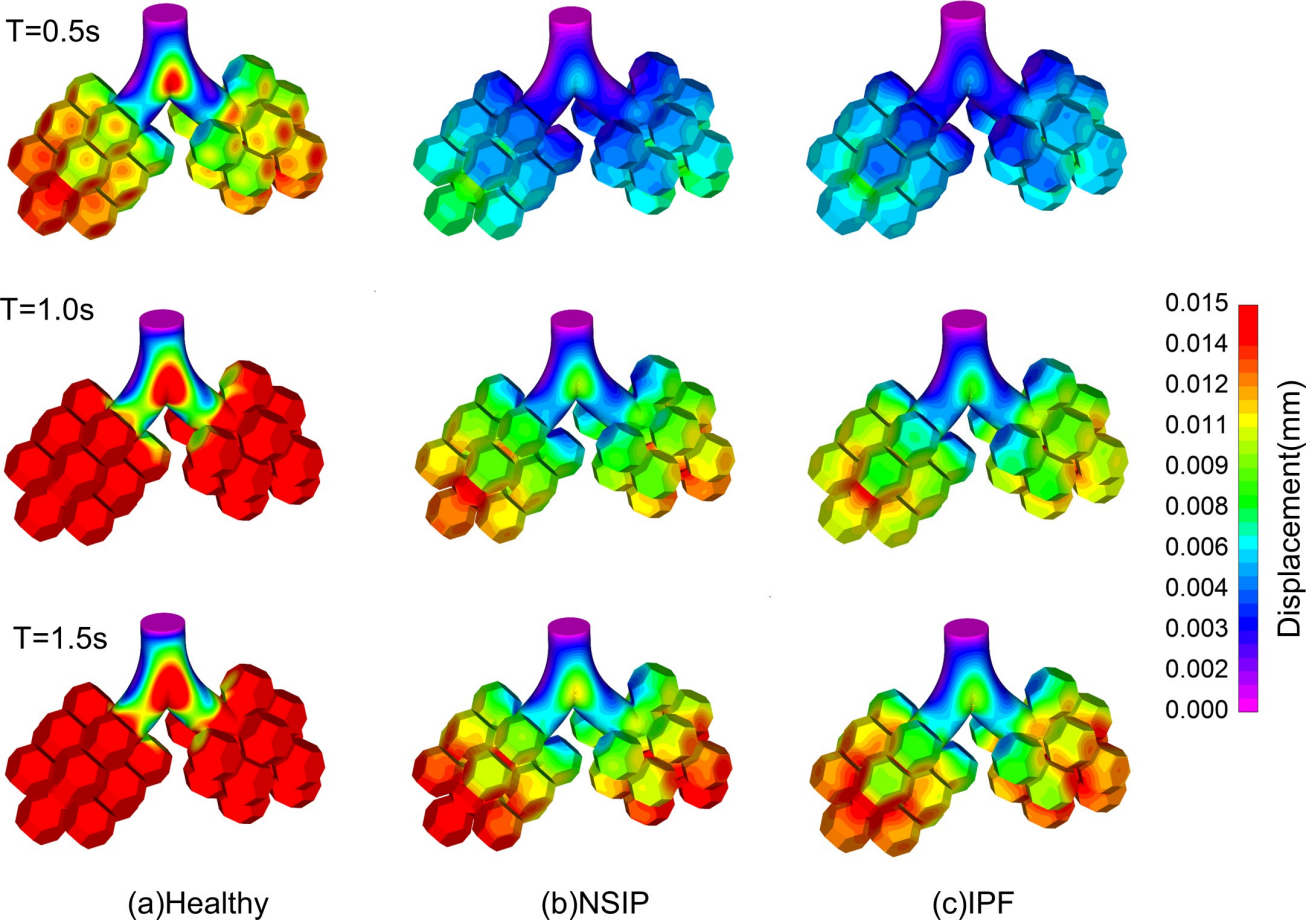
Temporal variation in contour of wall displacement at T = 0.5 s, T = 1.0 s, T = 1.5 s. (a) Healthy, (b) NSIP, and (c) IPF.

Fig 7 shows the contours of the wall displacement. The wall displacement represents the displacement of the inner surface of the tissue with respect to T = 0 s at each moment, and also the change in volume. The results show that the tissue has the largest displacement at the end of inspiration. The displacement in the healthy model is the largest. The displacement in NSIP is larger than that of IPF in the breathing cycle.

**Fig 8:**
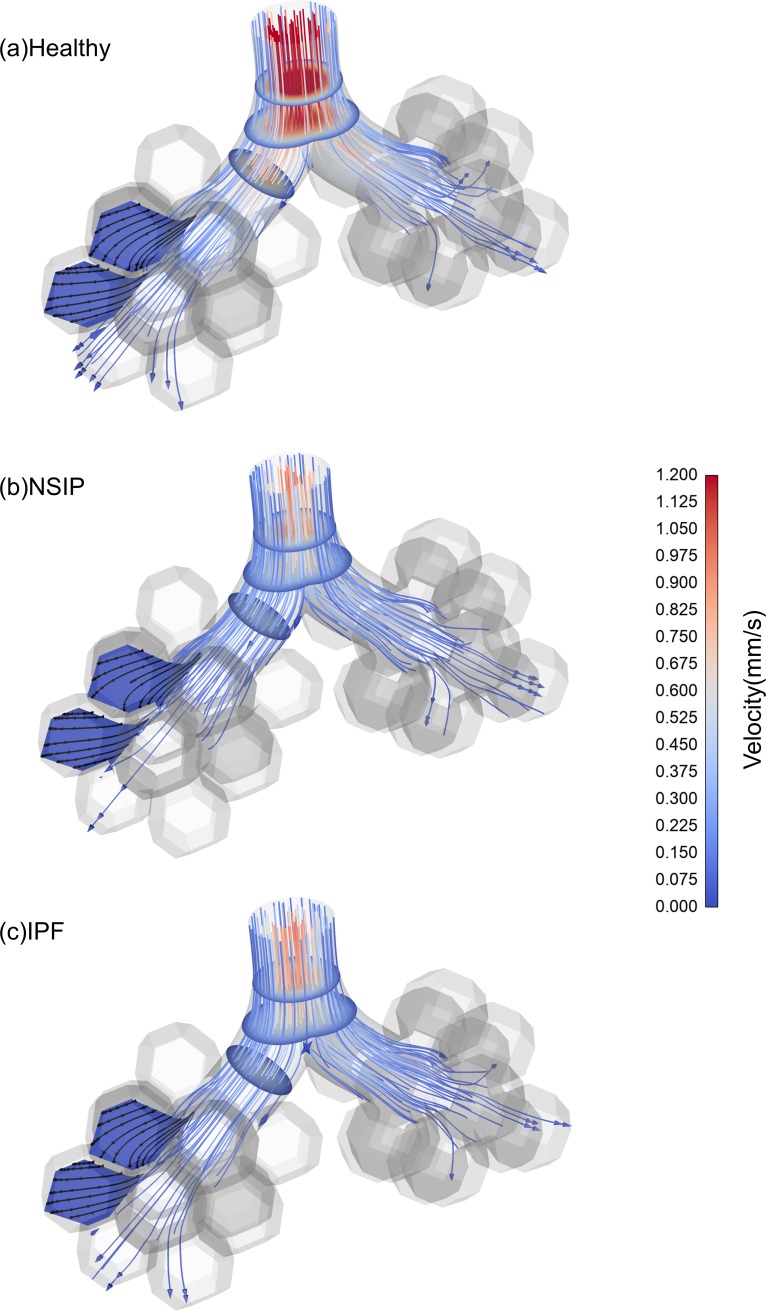
Flow velocity contours and streamlines at peak inspiration. (a) Healthy, (b) NSIP, and (c) IPF.

Fig 8 shows the streamlines and the contours of slices, which are colored by the velocity magnitude at peak inspiration. It is observed in the healthy model that the velocity magnitude is approximately 80% higher than that of the NSIP and IPF models. The maximum magnitude of the wall shear stress (WSS) is about 0.00162 Pa in the healthy model, while in the NSIP and IPF models, it is approximately 0.00086 Pa (as shown in S2 Fig).

**Fig 9:**
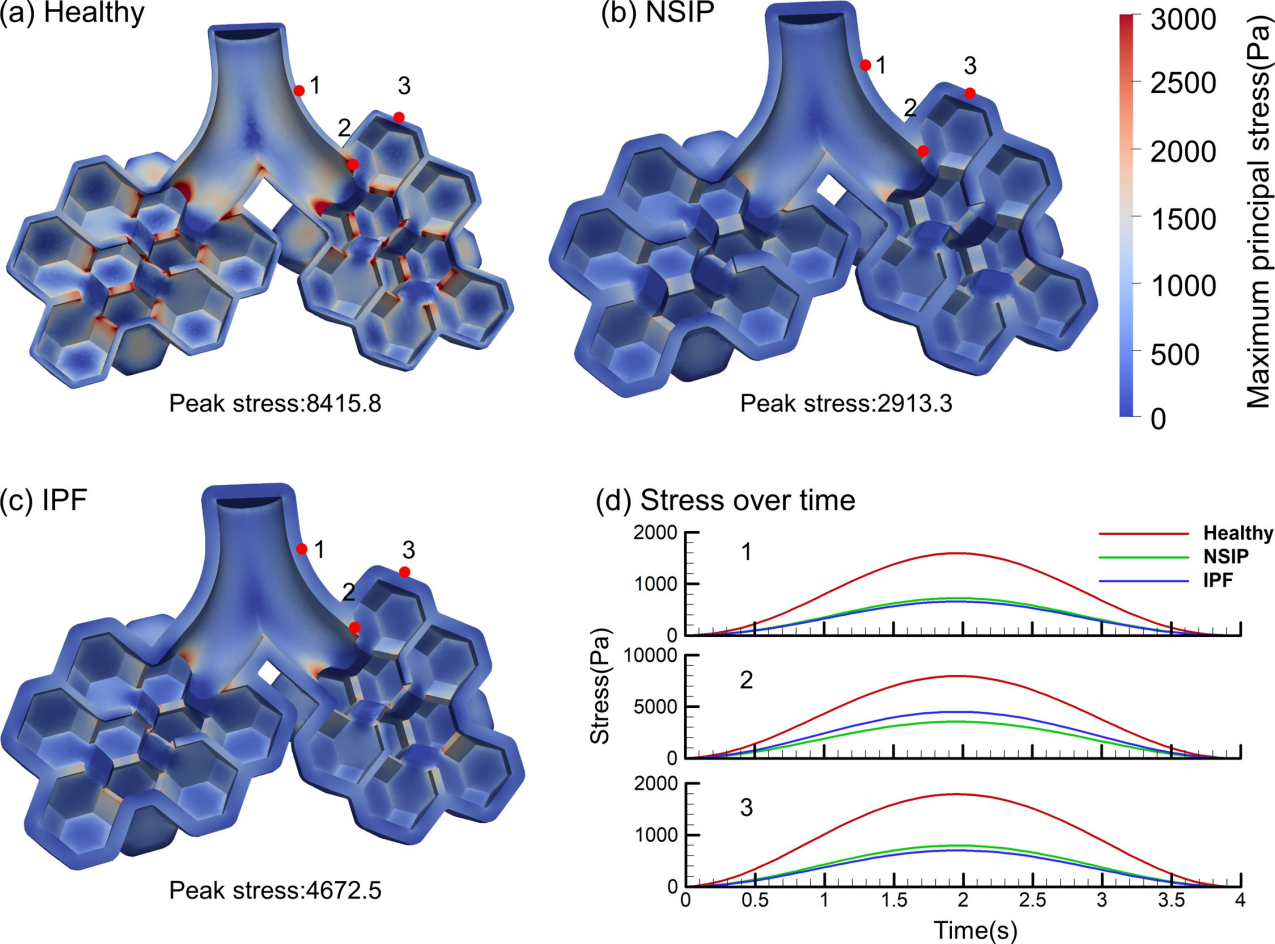
Stress contours of tissue at peak inspiration. (a) Healthy, (b) NSIP, and (c) IPF. (d) Stress over time at several positions.

The maximum principal stress in the pulmonary acinus at peak inspiration is presented in Fig 9. The secondary septa become overstretched compared to the primary septa. Thin regions near the junction of the alveolar sac and the trachea are the regions with the largest stress. The maximum principal stress of the NSIP and IPF models is smaller than that of the healthy model, because the stretch and volume changes of NSIP and IPF are smaller. The peak stress of the healthy, NSIP and IPF models is about 8415.8, 2913.3 and 4672.5 Pa respectively as shown in Fig 9A, 9B and 9C. Fig 9D shows the tissue stress over time at several positions. (The mean stress at these positions is shown in the S2 Table.)

### Pulmonary acinus function

**Fig 10:**
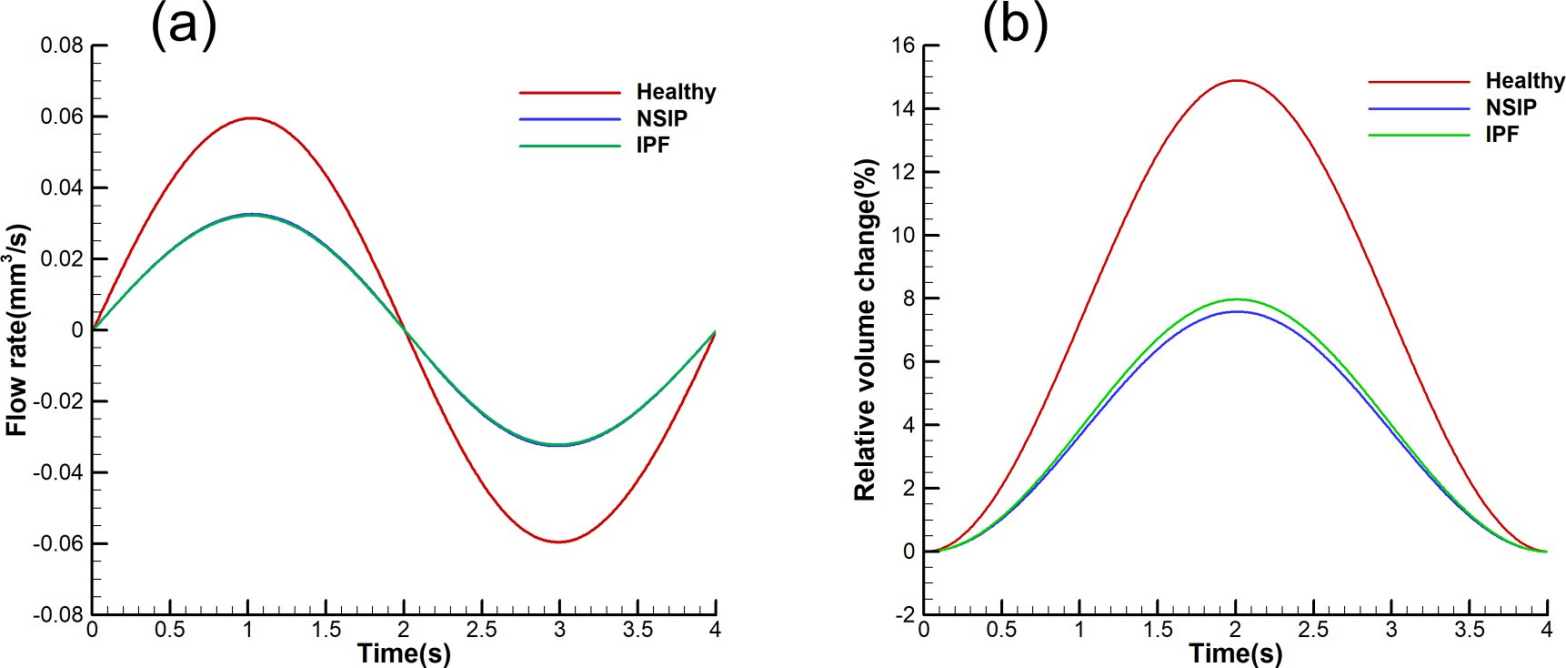
Comparison of mechanical characteristics of healthy, NSIP, and IPF models. (a) Comparison of the flow rate history. (b) Comparison of the flow domain relative to the volume change history.

A comparison of the flow rate history curve is presented in Fig 10A, and the flow domain relative to the volume change history curve is shown in Fig 10B. In the breathing cycle, the flow rate of the healthy model is significantly larger than that of the NSIP and IPF models. Similar trends are observed for the relative volume change. The flow rate of NSIP is almost the same as that of IPF. The maximum of the flow rate in the healthy model is approximately 0.059 mm^3^/s at peak inspiration, while in the NSIP and IPF models, it is approximately 0.032 mm^3^/s. The maximum relative volume change of IPF is slightly larger than that of NSIP. According to the maximum relative volume change and initial volume, the volume change of NSIP is 0.0374 mm^3^, and the volume change of IPF is 0.0371 mm^3^. This means that the TVs of NSIP and IPF are almost identical, which met the expectation of our model settings.

**Fig 11:**
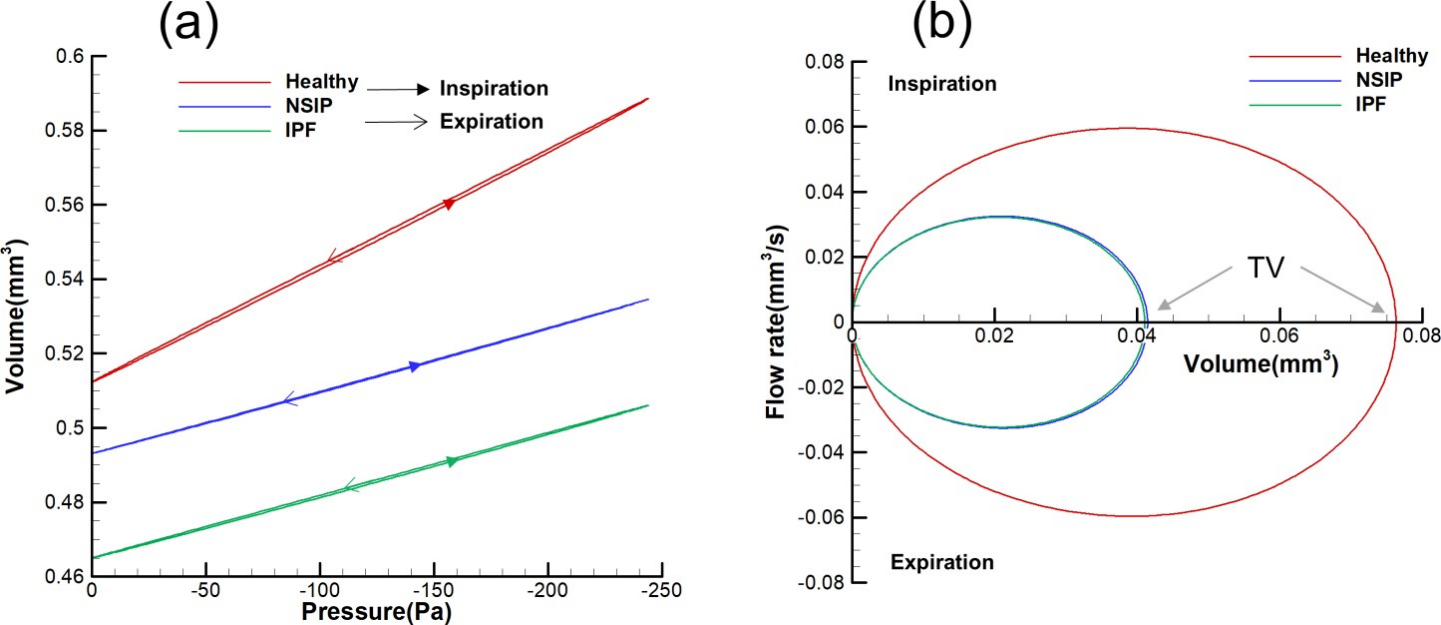
Comparison of lung function characteristics for healthy, NSIP, and IPF models. (a) Comparison of pressure-volume loops. (b) Comparison of flow-volume loops.

As shown in Fig 11A, the pulmonary acinus compliance (defined as the difference of volume divided by the difference of airway pressure) is represented by the slope of the pressure-volume (P-V) loop diagram. In NSIP and IPF, the pulmonary acinus compliance is lower than that of a healthy man; that is, for every 1 Pa change in pressure, the change in volume is less. There is a 47% decrease in pulmonary acinus compliance for NSIP and IPF compared with the healthy pulmonary acinus.

Fig 11B shows the comparison of flow-volume (F-V) loops. The results indicate that the inspiratory and expiratory parts of the F-V curve are symmetrical. The peak inspiratory flow (PIF) is equal to the peak expiratory flow (PEF). The TV of the healthy model is significantly different from the two disease models.

**Fig 12:**
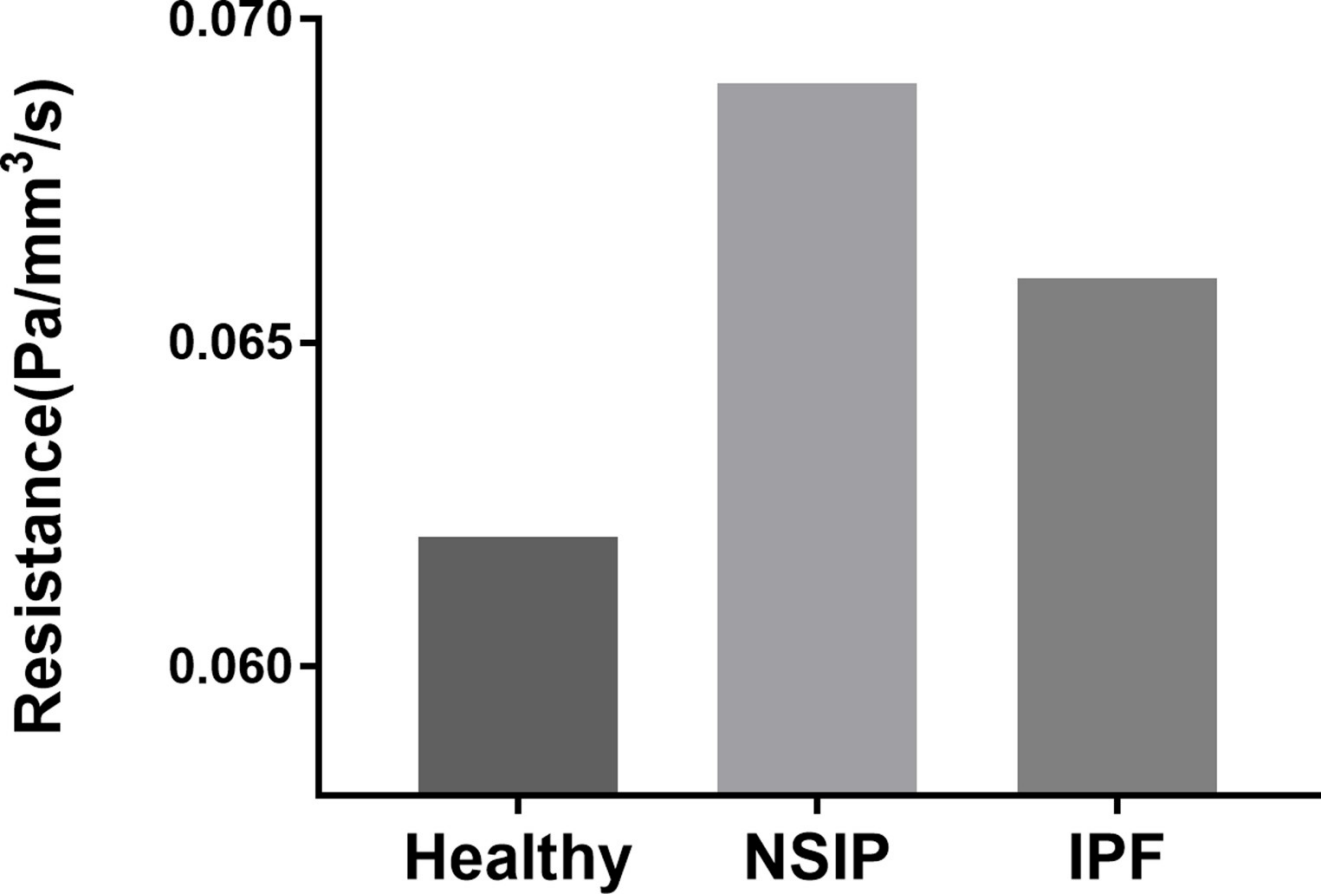
Comparison of pulmonary acinus resistance for healthy, NSIP, and IPF models.

As shown in Fig 12, the results of pulmonary acinus resistance (defined as the pressure divided by the airflow rate) among the healthy, NSIP and IPF models are shown in the histogram. The resistance of NSIP and IPF is higher than that of the healthy lung by 6.4~11.2%. In particular, the resistance of the NSIP lung is higher than that of the healthy IPF by 4.5%.

## Discussion and conclusion

One of the most important factors of IIP prognosis is based on a determination of the lung function with a typical restrictive ventilatory defect [40]. Establishing the early diagnosis of a distinct IIP and identifying the disease stage is paramount to improving the efficiency of patient treatment. Our research establishes computational 3D models of normal breathing in the healthy, NSIP, and IPF pulmonary acinus using FSI and compares the differences in the pulmonary function parameters among the three groups. A negative pressure is applied on the outside surface of the pulmonary acinus, which causes air to flow in and out of the pulmonary acinus, which is coincident with the negative pressure of the thoracic cavity in normal respiration and is physiologically accurate [41]. The maximum Reynolds number based on the alveolar sac lengths (1 mm) is 0.08 in the healthy model. Thus, the flow field is completely laminar. It is observed that the air flow in the distal alveolus is radial without recirculation, and the velocity is very low at the end of the alveolus. This observation is in good agreement with previous studies [42–45].

IIPs primarily involve the alveolar regions and show multiple alterations in lung physiology. IIPs typically identify a reduced forced vital capacity (FVC), a reduced total lung capacity, and a reduction in the diffusing capacity of the lung for carbon monoxide (DLCO) [2]. Studies indicate poor sensitivity of the lung volume measurements for the diagnosis of IPF [5, 46]. Furthermore, the pulmonary function test in the clinic, such as spirometry, is influenced by many factors, such as neuromuscular disorders [47]. In our research, the flow rate, volume change and maximum pressure drop of the healthy model are significantly larger than that of the NSIP and IPF models. The pressure drop decreases by 43% for the NSIP and IPF models compared with that of the healthy model. The maximum of the flow rate in the healthy model is approximately 0.059 mm^3^/s at peak inspiration, while in the NSIP and IPF models, it is approximately 0.032 mm^3^/s. The parameters we obtained from the 3D pulmonary acinus model show the differences of healthy, NSIP and IPF lung, which may provide some clues on identifying healthy, NSIP and IPF.

Pulmonary compliance is a measure of the lung's ability to stretch and expand [48]. Low compliance indicates a stiff lung, which is often seen in fibrosis. High compliance indicates a pliable lung, which is often seen in emphysema. Static lung compliance is perhaps the most sensitive parameter for the detection of abnormal pulmonary mechanics [49]. Our simulation results show that there is a 47% decrease in pulmonary acinus compliance for the NSIP and IPF in comparison with the healthy pulmonary acinus. Pulmonary compliance results can be normal when emphysema is also present, which can be investigated in future work.

Airway resistance is the resistance of the respiratory tract to airflow during inhalation and expiration. There are two main determinants of airway resistance, including the diameter of the airways and whether the airflow is laminar or turbulent. Airway resistance can be normal during the early stages of IIPs [50]. In our study, the results of pulmonary acinus resistance (defined as the pressure divided by the airflow rate) among the healthy, NSIP and IPF models are detected. The pulmonary acinus resistance of NSIP and IPF is higher than that of the healthy lung. In particular, the pulmonary acinus resistance of the NSIP lung is higher than that of IPF. The results may lay a foundation for further study in the early and differential diagnosis of IIPs.

There are three major limitations in this study that could be addressed in future research. First, the surface tension of the gas-liquid interface is not included in the model. Due to the complexity of CFD and FSI simulations, many CFD or FSI studies of airway do not contain surface tension [10–12, 36], just like our research. However, the surface tension contributed a large part of the hysteresis [39, 51] and facilitate anisotropic deformation on the alveolar surface [52]. In the future, the dynamic compression relaxation model for lung surfactants [53] could be included in the model. Second, the simulation is based on normal breathing. Deep breathing leads to large deformation of the alveolar surface, which is a challenge for the dynamic mesh method in Nalu code. In the future, immersed boundary method [9] could be employed for large deformation. Finally, the P-V and F-V loops in our simulation are different from those obtained in the spirometry because the simulation is based on normal breathing. The loops of the local pulmonary acinus are also different from those of the entire respiratory system.

One of the key advantages of FSI is the quantification of the impact of changes in the structure on flow properties. For future studies, patient-specific FSI models of the lung mean that there is a combination of medical imaging, such as CT with FSI. Future studies will inform us about further use of patient-specific FSI parameters as novel biomarkers in clinical trials and clinical practice-improved predictions and assessments of clinical treatment.

In conclusion, this study provides a qualitative description of how air flow and the pulmonary acinus function are affected by NSIP and IPF. The tissue of NSIP and IPF models become stiffer, and pulmonary compliance decreases compared with that of healthy models. In particular, the pulmonary acinus resistance of the NSIP lung is 4.5% higher than that of IPF, which is a sensitive lung function parameter for IIP. The potential applications of the present work to the clinical situation may be further studied on individual image-based FSI simulations for earlier diagnosis and assessment of disease in the future.

## Supporting information

S1 File Pulmonary acinus geometry for the healthy, NSIP and IPF models. (ZIP)

S2 File Example of the Nalu solver input file. (INPUT)

S3 File Python script for top level FSI iterations. (PY)

S1 Fig Comparison of relative volume change at different time steps. (TIF)

S2 Fig Wall shear stress distribution at peak inspiration. (TIF)

S1 Table Mesh convergence study parameters. (DOCX)

S2 Table Mean stress of tissue at several positions. (DOCX)
