# Supplementary figures and images for: Characterization of air flow and lung function in the pulmonary acinus by fluid-structure interaction in idiopathic interstitial pneumonias
Source: PLoS One. 2019 Mar 28;14(3):e0214441. doi: 10.1371/journal.pone.0214441 (PMC6438611; doi:10.1371/journal.pone.0214441)

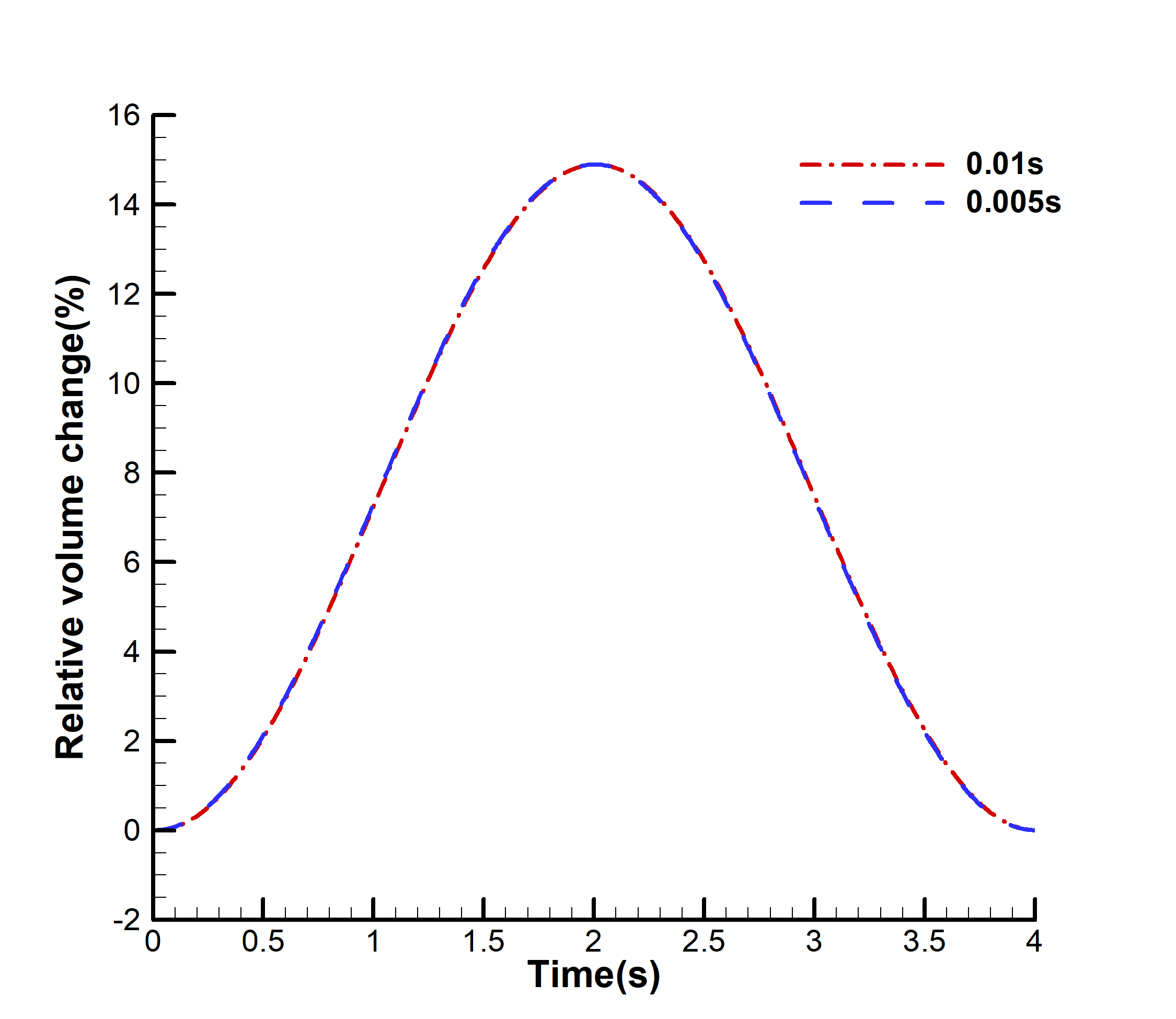

Supplement: S1 Fig — (TIF) [file pone.0214441.s004.tif]

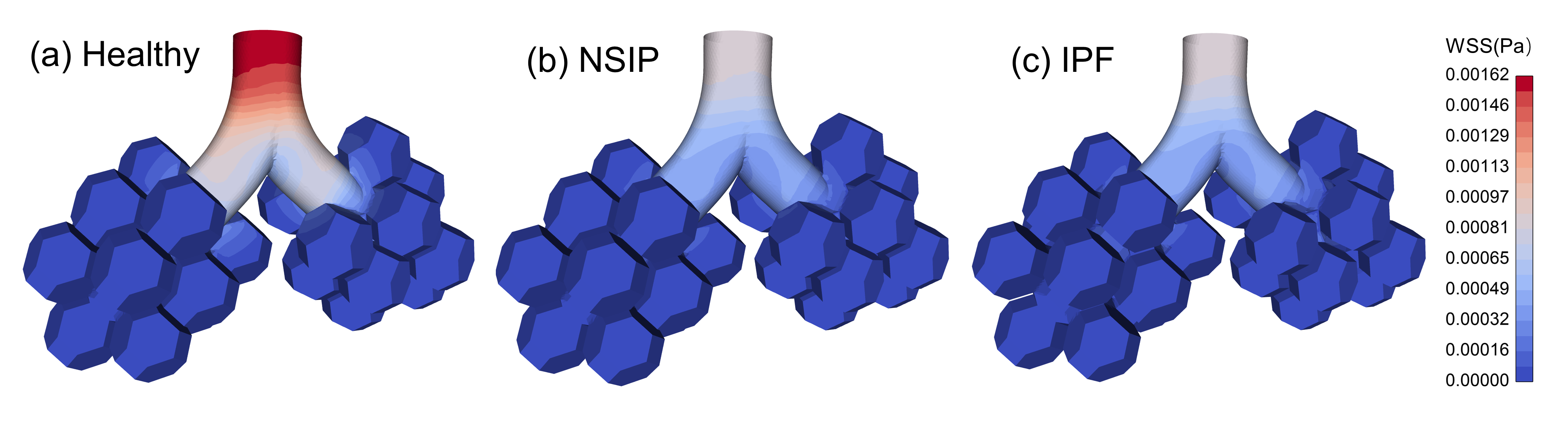

Supplement: S2 Fig — (TIF) [file pone.0214441.s005.tif]
