# Supplementary material for: Characterization of air flow and lung function in the pulmonary acinus by fluid-structure interaction in idiopathic interstitial pneumonias
Source: PLoS One. 2019 Mar 28;14(3):e0214441. doi: 10.1371/journal.pone.0214441 (PMC6438611; doi:10.1371/journal.pone.0214441)
Supplement: S1 Table — (DOCX) [file pone.0214441.s006.docx]

**S1 Table. Mesh convergence study parameters.** Three different mesh sizes ranging from 1.34M to 3.92M CFD mesh elements and 0.74M to 1.37M CSD mesh elements are generated for healthy model. Mesh convergence study parameters are shown with the flow rate at peak inspiration being the monitored parameter. The flow rate at peak inspiration changed by only 0.33% (less than 1%) between medium and fine meshes. The medium meshes achieves suitable accuracy and is used for subsequent simulations. The meshes for the NSIP and IPF models are generated using the same element size as medium meshes for healthy model.

| Mesh | CFD mesh elements | CSD mesh elements | Flow rate at peak inspiration (mm^3^/s) |
| --- | --- | --- | --- |
| Coarse | 1.34M | 0.74M | 0.05870 |
| Medium | 2.31M | 0.99M | 0.05938 |
| Fine | 3.92M | 1.37M | 0.05979 |
