# Supplementary material for: Characterization of air flow and lung function in the pulmonary acinus by fluid-structure interaction in idiopathic interstitial pneumonias
Source: PLoS One. 2019 Mar 28;14(3):e0214441. doi: 10.1371/journal.pone.0214441 (PMC6438611; doi:10.1371/journal.pone.0214441)
Supplement: S2 Table — (DOCX) [file pone.0214441.s007.docx]

**S2 Table. Mean stress of tissue at several positions.** The details of these positions are shown in Fig 9.

| Mean stress(Pa) | Healthy | NSIP | IPF |
| --- | --- | --- | --- |
| Position 1 | 798.0 | 362.6 | 330.1 |
| Position 2 | 3986.2 | 1777.0 | 2253.1 |
| Position 3 | 896.3 | 399.4 | 351.7 |
